# Supplementary figures and images for: gmos: Rapid Detection of Genome Mosaicism over Short Evolutionary Distances
Source: PLoS One. 2016 Nov 15;11(11):e0166602. doi: 10.1371/journal.pone.0166602 (PMC5112998; doi:10.1371/journal.pone.0166602)

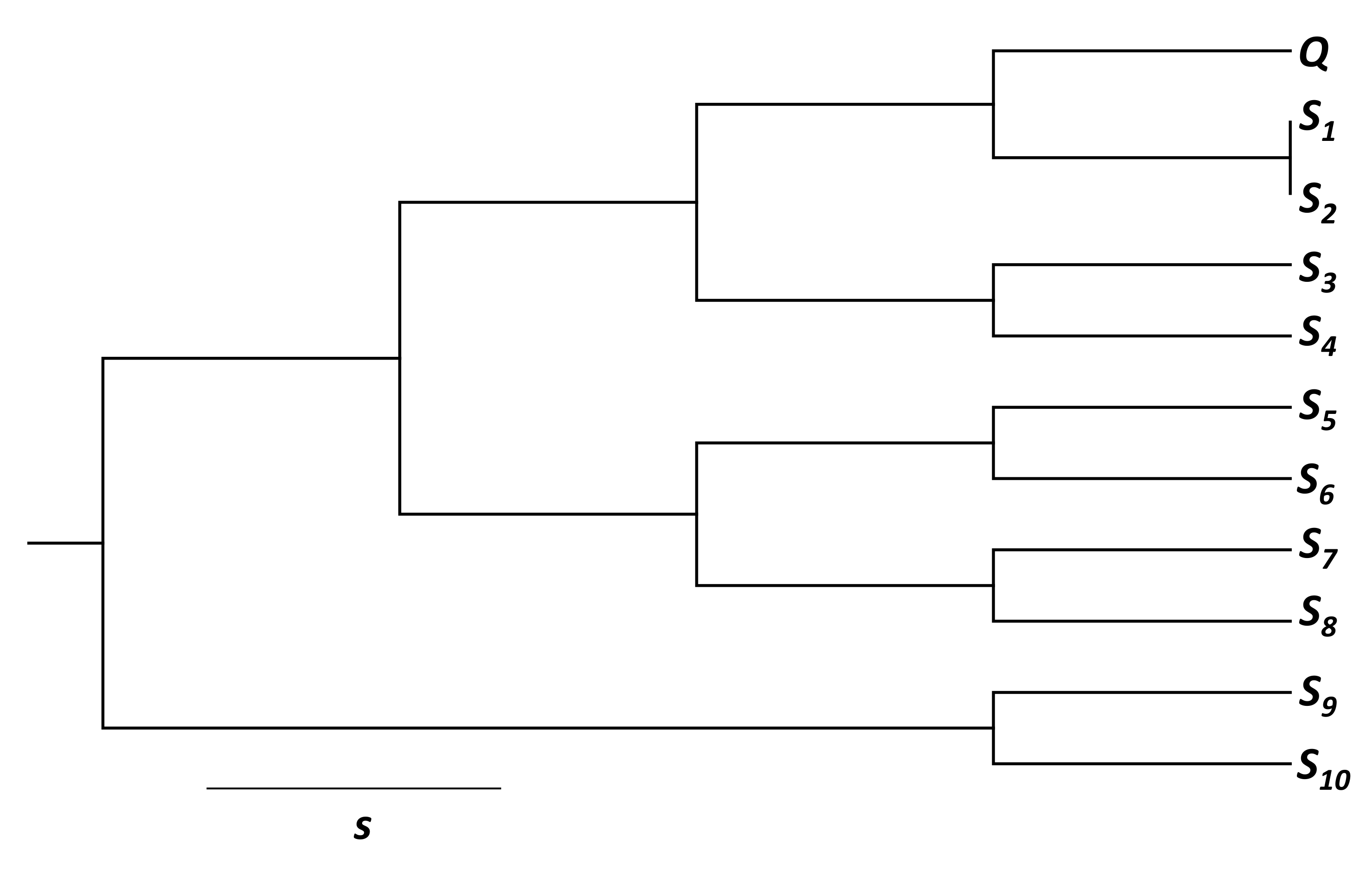

Supplement: S1 Fig — Genealogy of a query sequence Q and the subject set {S1, S2, …, S10} (simulated data). Q is most closely related to sequences S1 and S2 along its entire genome. (TIF) [file pone.0166602.s001.tif]

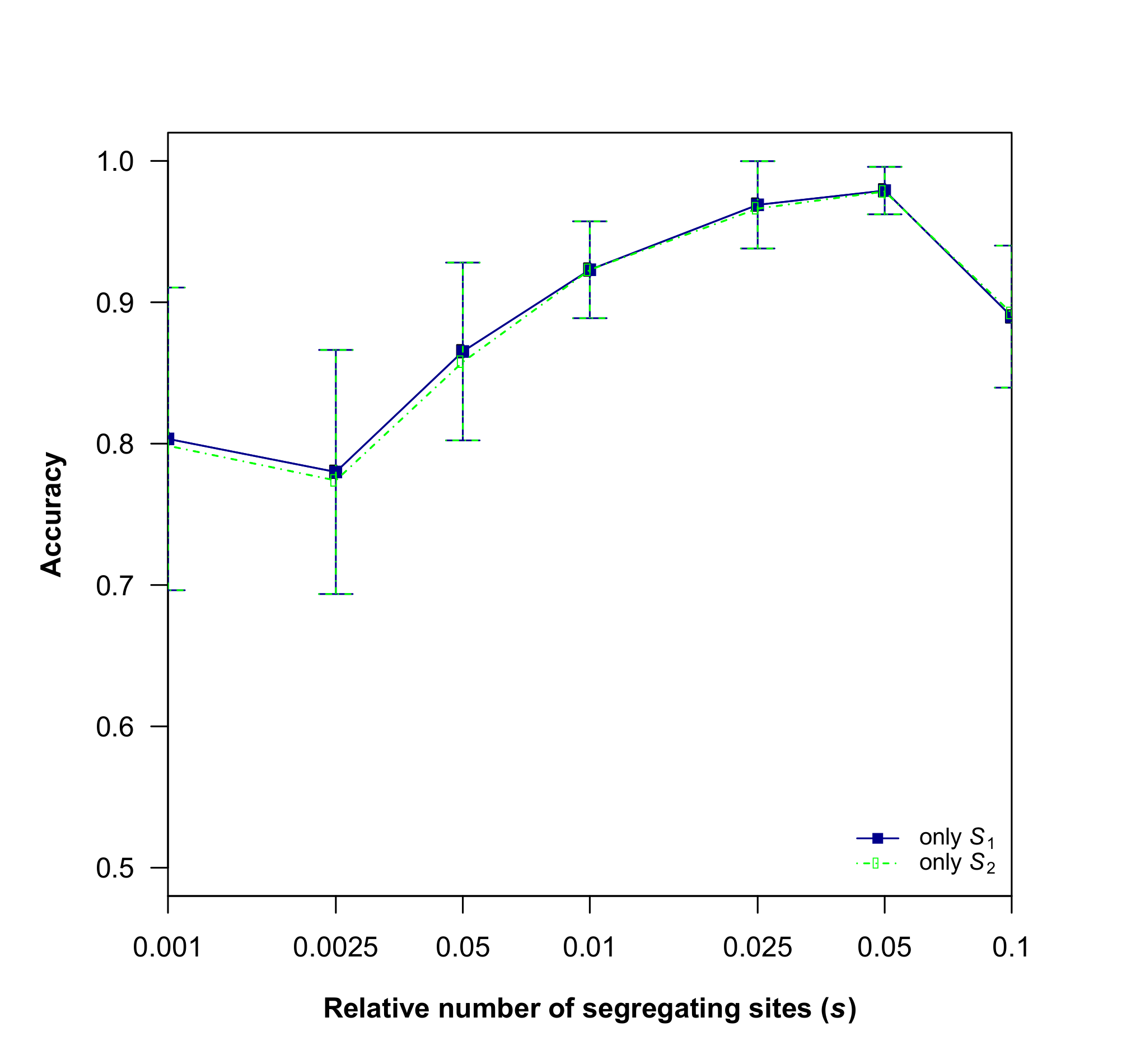

Supplement: S2 Fig — Proportion of correctly returned subject sequences S1 and S2 using the simulated genealogies (S1 Fig) as a function of the evolutionary distance (the relative number of segregating sites), s. (TIF) [file pone.0166602.s002.tif]
