# Supplementary material for: gmos: Rapid Detection of Genome Mosaicism over Short Evolutionary Distances
Source: PLoS One. 2016 Nov 15;11(11):e0166602. doi: 10.1371/journal.pone.0166602 (PMC5112998; doi:10.1371/journal.pone.0166602)
Supplement: S1 File — The results of accuracy and speed comparison of gmos to ClonalOrigin on simulated data sets. (PDF) [file pone.0166602.s003.pdf]

Table A – Comparison of *gmos* to *ClonalOrigin*

	**gmos**			**ClonalOrigin**			
s	***TPR* [%]**	***FPR* [%]**	***t* [s]**	***TPR* [%]**	***FPR* [%]**	***Number of detected RE***	***t* [s]**
0.001	56.73	19.71	0.03	17.68	5.56	1.20	1861.55
0.0025	67.07	9.38	0.03	58.48	7.78	1.90	1941.84
0.005	85.63	11.30	0.02	90.36	6.03	2.10	1884.47
0.01	83.50	10.00	0.02	**91.78**	**5.04**	**2.00**	**2581.88**
0.025	82.42	6.90	0.02	98.37	11.24	2.80	3924.46
0.05	94.59	5.18	0.02	44.90	17.90	9.50	12231.2
**0.1**	**94.42**	**1.64**	**0.03**	*			
0.11	92.63	2.82	0.04	*			

Comparison of gmos to ClonalOrigin on simulated data sets, where each data set includes a recombinant query sequence, whose genealogy is shown in Fig 3 (see text for the details). The parameters *TPR* (the true positive rate), *FPR* (the false positive rate), *Number of detected RE* (the number of recombination events detected by ClonalOrigin) and *t* (the running-time measured in seconds) were calculated for each value of *s* (the evolutionary distance calculated as the relative number of segregating sites; Fig 3). The parameters' values were computed as the average over 10 data sets. In case of ClonalOrigin, *TPR* and *FPR* were measured for the recombination events, where S_2 was the donor sequence and the query was the recipient: *TPR* is the fraction of query positions correctly detected to be horizontally transferred from the sequence S_2 and *FPR* is the fraction of query positions incorrectly detected to be horizontally transferred from the sequence S_2. In case of gmos, *TPR* and *FPR* were measured for query positions assigned to S_2, where S_2 was detected as the best hit: *TPR* is the fraction of query positions correctly assigned to be most closely-related to S_2 and *FPR* is the fraction of query positions incorrectly assigned to be most-closely related to S_2.

*Due to decrease in performances and expensive computation, ClonalOrigin was not tested for $s \geq 0.1$. The rows with bolded values represent the best scores for gmos (s=0.1) and ClonalOrigin (s=0.01), respectively.
